# Epidemiological assessment and therapeutic response in hypopharyngeal cancer

**DOI:** 10.5935/1808-8694.20130089

**Published:** 2015-10-08

**Authors:** Ali Amar, Otávio Alberto Curioni, Diógenes Lopes de Paiva, Abrão Rapoport, Rogério Aparecido Dedivitis, Claudio Roberto Cernea, Lenine Garcia Brandão

**Affiliations:** a PhD in Otorhinolaryngology and Head and Neck Surgery, Federal University of São Paulo - UNIFESP (Assistant Physician - Department Head and Neck Surgery and Otorhinolaryngology - Heliópolis Hospital).; b Senior Associate Professor - Department of Surgery - School of Medical Sciences - Lusiada Foundation, Santos/SP (Head of the Department of Head and Neck Surgery and Otorhinolaryngology - Heliópolis Hospital, São Paulo. Head and Neck Surgeon of São José Hospital - RBBP, São Paulo).; c Resident physician in the Department of Head and Neck Surgery and Otorhinolaryngology, Heliópolis Hospital (Resident Physician, Department of Head and Neck Surgery and Otorhinolaryngology, Heliópolis Hospital).; d Senior Associate Professor at the Department of Surgery, Medical School of the University of São Paulo (Technical Director of the Department of Health, Heliópolis Hospital, São Paulo. Head and Neck Surgeon - RBBP of São José Hospital, São Paulo).; e Senior Associate Professor - Larynx Group Supervisor - Department of Head and Neck Surgery, Medical School of the University of São Paulo (MD).; f Associate Professor, Department of Head and Neck Surgery, Medical School of the University of São Paulo.; g Full Professor - Department of Head and Neck Surgery, Medical School of the University of São Paulo. Department of Head and Neck Surgery and Otorhinolaryngology, Heliópolis Hospital, São Paulo, Brazil.

**Keywords:** epidemiology, hypopharyngeal neoplasms, neoplasm staging, squamous cell carcinoma, survival analysis

## Abstract

Despite the low incidence, diagnostic and therapeutic advances, hypopharyngeal cancer still has high mortality.

**Objective:**

To evaluate retrospectively the epidemiological profile and response to surgery and radiation/chemotherapy of patients with hypopharyngeal cancer.

**Method:**

We reviewed the medical records of 114 patients treated between 2002 and 2009 in a tertiary hospital with histopathological diagnosis of squamous cell carcinoma.

**Results:**

The mean age of the patients was 57 years, 94.7% were males and 5.3% females, 98.2% were smokers and 92% consumed alcohol; 72% are illiterate or did not complete first grade schooling. The main complaints were: neck node (28%), pain and dysphagia (22%), odynophagia (12.2%), dysphonia (7.8%). The clinical staging was: I (1.7%), II (3.5%), III (18.4%), IV (76.3%). The treatment was carried out with radiotherapy and chemotherapy alone in 35%, with mean 2-year survival of 20% and 5-year survival of 18%; surgery followed by radiotherapy and chemotherapy in 22.8% with 2-year survival of 60.0% and 5 years of 55.0%; chemotherapy alone in 2.6%, and 39.4% without treatment.

**Conclusion:**

Most patients already had advanced clinical stages and independent of the treatment option, had a low survival rate, confirming the poor prognosis of this neoplasm.

## INTRODUCTION

Squamous cell carcinoma of the hypopharynx is relatively rare and bears the worst prognosis of all head and neck tumors, particularly those located in the piriform recess which is the most frequent site of origin in the hypopharynx^1^, ^2^. According to U.S. data from the National Cancer Data Base, this condition corresponds to 4% of all head and neck tumors and 7% of all malignancies of the upper aerodigestive tract^3^. According to national data from the Brazilian INCA, it accounts for 30% of pharyngeal tumors, it predominates in males and 80% are located in the piriform recess^4^. Among the major risk factors we list smoking and alcohol consumption, which separately have specific and significant effects in the hypopharynx, besides having a statistically significant synergistic effect according to numerous authors^5^; and as for complementary risk factors we have sideropenia and syphilis^4^.

There is a high incidence of advanced cases already found upon initial presentation compared to other sites of the head and neck, such as the larynx^6^, having distant metastases, and especially regional metastases - which may be the first clinical manifestation of the disease^7^. Data from the Department of Health shows that 23% of patients are in clinical stage III and 72% in stage IV^4^ - which causes these patients to have a median five-year survival by disease stage of 56% and 32%, respectively^4^.

There are many reasons for this poor prognosis: the hypopharynx is a silent area, patients usually have a bad overall clinical and nutritional status, submucosal spread is characteristic in these cases with known likelihood of developing regional and distant metastases, and capacity for direct invasion of adjacent organs of the head and neck^8^. Treatment decision is impaired because of the aforementioned prognosis, low nutritional intake upon admission and the high occurrence of second primary tumors, which together lead to a considerable reduction in overall survival^9^.

In our clinic, the standard treatment is carried out by partial surgery or radiation therapy for T1 and T2 cases, and pharyngolaryngectomy for T3 and T4 cases with a reserved preservation protocol for cases with a functional larynx. Because of disease-inherent factors and its carrier, and the aforementioned therapeutic weapons available, decisions are mainly based on: administering more or less powerful chemotherapeutic agents; carry on extended field or narrow field radiotherapy, and perform an extensive surgical resection, followed by the necessary reconstruction or a less extensive resection^10^.

Still talking about treatment, it should be noted that despite the availability of more potent chemotherapeutic agents in the market, more targeted radiotherapy modalities, improvements in surgical techniques, and especially the possibilities of reconstruction, more accurate imaging and better understanding of the pathophysiology of this disease^10^, we could not translate these advances into improved overall survival, confirming the difficulty in selecting the best treatment for each case.

The objective of this study is to evaluate the epidemiology of the patients admitted to a tertiary hospital with a diagnosis of squamous cell carcinoma of the hypopharynx, its relationship with the risk factors, as well as an analysis of survival according to treatment.

## METHOD

This is a retrospective study of patients with squamous cell carcinoma of the hypopharynx treated between 2001 and 2009 in a tertiary hospital with histopathological confirmation, and we took off tumors of other sites extending to the hypopharynx, as well as suspicious lesions and second tumors in this region without pathologic confirmation.

This study was approved by the Ethics Committee of the Institution where it was performed under protocol No. 71/2000.

## RESULTS

We selected 114 patients with squamous cell carcinoma of the hypopharynx. The patients’ ages ranged from 38 to 81 years, with mean age of 57 years, occurring predominantly in males, with 108 men (94.7%) and only eight women (5.3%) in our sample.

The chief complaint at presentation was a cervical node in 28% of cases, followed by pain and dysphagia (22%), sore throat (12.2%), dysphonia (7.8%) and foreign body sensation (6.1%) and there may be an initial manifestation of more than one concomitant symptom. The mean time between the onset of the chief complaint and admission to the clinic was 5.4 months (ranging between 1 and 24 months).

Regarding the clinical stage at initial presentation: 1.7% was in stage I; 3.5% in stage II; 18.4% stage III; and 76.3% were in stage IV. Among the major risk factors, we found that 98.2% were smokers and 92% were alcoholics. With respect to educational level, 72% were illiterate or had incomplete basic education.

**Figure 1:**
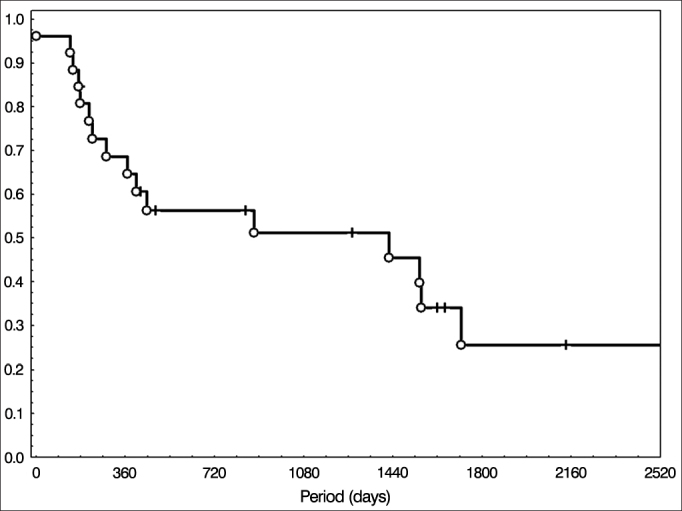
Disease-free survival in patients treated with surgery followed by radiotherapy.

**Figure 2:**
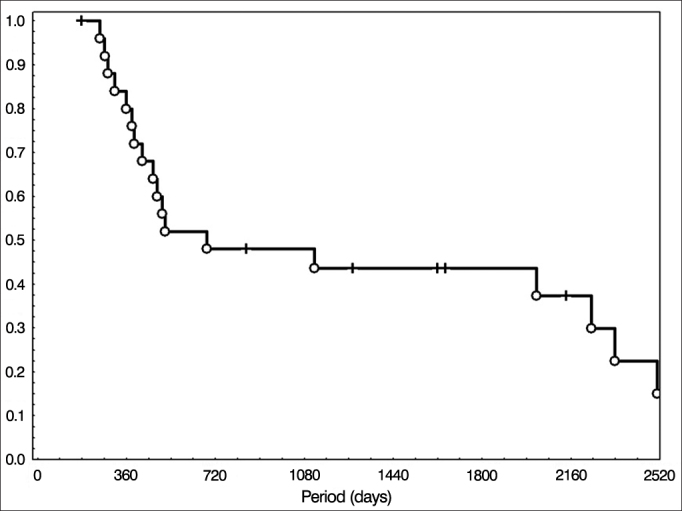
Overall survival of the patients treated with surgery followed by radiotherapy.

Regarding treatment, surgery was the initial choice in 26 patients (22.8%) followed by radiotherapy. The disease-free and overall survival curves of this group were traced using the Kaplan-Meier method and are presented in Figures 1 and 2, respectively. The mean two-year overall survival in this group was 47.5%, and 37.5% in five years.

**Figure 3:**
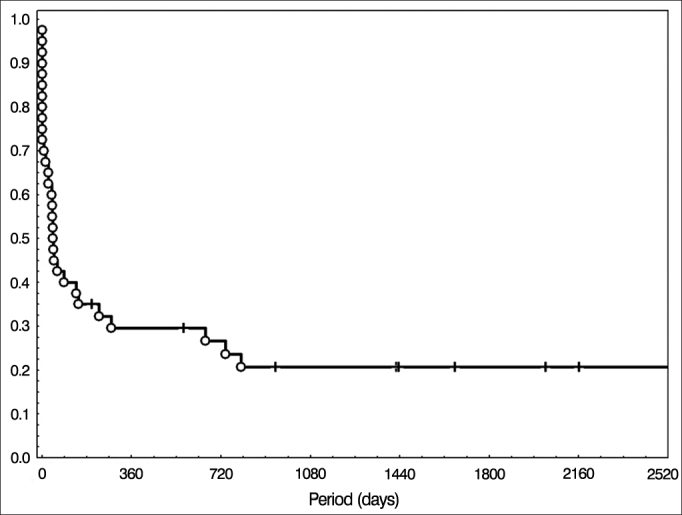
Disease-free survival in the group treated with radiotherapy and chemotherapy.

**Figure 4:**
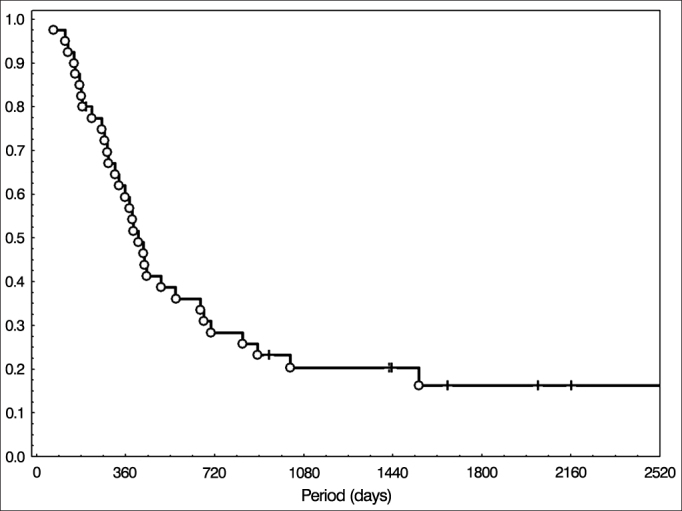
Overall survival in the group treated with radiotherapy and chemotherapy.

Concomitant radiotherapy and chemotherapy was the initial treatment of choice in 40 patients (35%) and disease-free and overall survival and overall curves of this group were also traced following the Kaplan-Meier method and are depicted in Figures 3 and 4, respectively. Overall survival at two years was 40%, and 20% in five years.

Palliative chemotherapy was performed in three patients. Forty-five patients (39.4%) were untreated. Of this latter group, 18 died before the start of treatment or did not have medical conditions to withstand any form of therapy and 27 did not return after the first visit or after referral to radiotherapy.

## DISCUSSION

This was a retrospective study carried out in one single institution, which evaluated patients with confirmed diagnosis of epidermoid carcinoma of the hypopharynx. Regarding the epidemiological data presented, there is an agreement with the current literature in relation to male predominance and a higher prevalence in adults older than 45 years^3^. Currently, there is an increase in the incidence of this disease in women, although its behavior and prognosis are similar to cases involving men. Regarding age, its later presentation correlates with the long history of exposure to the major carcinogens - tobacco and alcohol, are also consistent with current epidemiological data^5^.

The prevalence of the initial symptoms and their distribution depict some anatomical and behavioral peculiarities of the disease because initial tumors can grow inside the piriform recess without causing significant obstructive symptoms, thus leading patients to only show late symptoms of aerodigestive tract obstruction of the aerodigestive tract and pain arising from the neural infiltration^2^, as well as the patterns of submucosal spread of the lesions associated with the rich lymphatic network which cause early neck metastasis^11^, shown in the report results and identification of neck nodes as the first sign and most frequent symptom (28%).

Due to the large lymphatic spread and the silent character of the early cases previously reported, there is a high incidence of diagnosis of advanced clinical stages, showing, according to our data, 94.7% already in stages III and IV, contributing to the poor disease prognosis and survival.

Because of the high incidence of advanced cases, treatment is usually performed in combination - surgery followed by radiotherapy or radiotherapy and concomitant chemotherapy. Regarding surgery, it may be a partial pharyngectomy, done in selected cases due to the intimate contact of that region with the larynx and the need for resection with oncologic margin encompassing this region, performed in our series in only one patient via pharyngotomy. The most commonly used approach is pharyngolaryngectomy with or without flap reconstruction of the digestive tract, depending on the involvement of the posterior wall of the hypopharynx and, lastly, pharyngolaryngoesophagectomy in cases of cervical esophagus involvement, requiring reconstruction with jejunum or making a gastric tube.

Neck dissection may complement the surgery, usually held together because of the high incidence of neck metastasis. Contralateral neck dissection is indicated in some patients, especially in cases of positive ipsilateral neck and lesions that cross the midline, they add a lower rate of regional recurrence without, however, significantly altering overall survival^12^. It is worth considering that, regardless of the treatment paradigm, better results were not detected.

In our series, surgery was used as initial therapy in 22.8% of cases, leading to a mean two-year survival of approximately 50% with stabilization and a new drop at five years - about 40%. Considering the generally poor nutritional status of these patients, other comorbidities they have, possible complications that a major surgery can produce in such circumstances coupled with the long recovery and rehabilitation process, and the low overall survival, we must consider each individual case when recommending such intervention, even though it is option with the best results in cancel control^13^ and be the treatment of choice in our clinic.

Radiotherapy was the initial treatment choice in 35% of the cases, always with chemotherapy that enhances the effect of the first treatment. In some of our patients, we used a neoadjuvant chemotherapy regimen, often with cisplatin and 5-fluorouracil - although not changing survival in some studies^14^, it may be predictive of response to radiotherapy and chemotherapy. Even in the absence of partial response, it signals a poorer response to radiotherapy and quimioterapia^15^ and leads to modify the proposal for a surgical intervention. Regarding survival of this group, we have approximately 25% in two years and 20% in five years - a worse outcome when compared to the surgery group initially.

These results may stem from a selection bias, because as our protocol for advanced cases is surgery followed by radiation therapy and most patients already present with stages III and IV, we tend to refer to radiotherapy patients that have some criteria for tumor unresectability, such as invasion of the prevertebral fascia or major involvement of the internal carotid; or those who have comorbidities that contraindicate surgery, and other comorbidities such as malnutrition that often accompany these patients - which should not be an absolute contraindication to surgery^16^, but without proper support and multidisciplinary management it brings unacceptable risks to this procedure.

Considering some of the characteristics already mentioned inherent to patients and the tumor, three patients underwent palliative chemotherapy with no further treatment and 45 patients went without treatment - a total of 39.4% of our sample, further corroborating the aggressiveness of the disease, and the multiplicity of factors involved in the decision to treat or not these patients and last treatment possibility that could be more effective and less harmful for this group.

Another important factor is the socio-economic level and family support of patients, mostly living in poverty and unable to maintain minimum hygiene, supportive care shown to depend on the treatment, as well as their compliance towards it, and this another item to be considered in the management of these cases and also in impacting on the success of the treatment chosen^17^.

## CONCLUSION

Considering the high mortality of this tumor, the frequency of advanced disease at initial presentation and major morbidity of both forms of treatment available, the therapeutic decision of the hypopharynx carcinoma remains a challenge. Due to the need for multifactorial evaluation of these patients, an individual analysis should be carried out and one should consider the experience of the surgical team, and the availability of resources so that the best therapeutic choice can be made for each case.
